# Recent emergence of a novel porcine pestivirus: interference with classical swine fever diagnosis?

**DOI:** 10.1038/emi.2017.5

**Published:** 2017-04-12

**Authors:** Alexander Postel, Denise Meyer, Anja Petrov, Paul Becher

**Affiliations:** 1 EU & OIE Reference Laboratory for Classical Swine Fever, Institute of Virology, Department of Infectious Diseases, University of Veterinary Medicine, 30559 Hannover, Germany

**Dear Editor,**

One of the most important viral diseases of swine is classical swine fever (CSF), which causes tremendous losses in pig production worldwide and is, therefore, notifiable to the World Organization for Animal Health (OIE). In many countries worldwide, the CSF situation is monitored by PCR-based genome detection and serological screening to demonstrate the absence of the disease. The causative agent of CSF, classical swine fever virus (CSFV), is a single-stranded RNA virus that belongs to the genetically highly variable genus *Pestivirus* within the family *Flaviviridae*. Infections of pigs with other pestiviruses occasionally occur and need to be carefully ruled out because having a CSF-positive status has a great impact on a country's ability to engage in international trade of pigs and pig products.

Feto-pathogenicity is a common feature of pestivirus infections that occurs during gestation. The infection of gravid sows with low-virulent CSFV isolates can result in the birth of piglets with neurological disorders. This clinical syndrome is designated congenital tremor (CT) or *Myoclonia congenita*.^1^ Very recently, it was reported that a newly discovered atypical porcine pestivirus (APPV) is also associated with CT in newborn piglets.^2, 3, 4^ Consequently, APPV-associated CT must be considered in the clinical differential diagnosis of CSF. Against this background, it is of utmost importance to determine whether the presence of this porcine pestivirus interferes with established procedures for CSF diagnosis. In this study, both the virological and serological differentiation of CSFV and APPV were addressed by applying well-established routine diagnostic methods and newly developed assays.

APPV genome-positive samples (*n*=14) obtained from different regions in Germany and Italy that contained ample amounts of APPV RNA (quantitation cycle (Cq) values between 16 and 35) were tested with three established quantitative real-time PCRs with reverse transcription (qRT-PCRs) that are routinely performed at the EU and OIE Reference Laboratory for CSF. These tests comprised one pestivirus-specific qRT-PCR^5^ and two CSFV-specific qRT-PCRs.^6, 7^ No false-positive results were obtained with these qRT-PCRs (Supplementary Table S1). However, it was possible to demonstrate that a newly designed primer pair (PanPesti_140f: 5′-AAG TCC YGA GTA CRG GRC AG-3′ and PanPesti_241r: 5′-TTG GGC ATG CCC WCG TCC AC-3′) that targets the 5′ non-translated region (NTR) of the pestiviral genome was broadly reactive with APPV and different CSFV genotypes. This result highlights that in the 5′ NTR, cross-reactivity can occur. Nevertheless, this broadly reactive SYBR Green based ‘PanPesti' qRT-PCR was less sensitive for most of the samples (11/14) than the previously described APPV-specific PCR (Supplementary Table S1). Apart from this newly developed assay, no cross-reactivity between CSFV and APPV genome detection was identified following the application of the methods that are routinely used for CSFV diagnosis. To assess the influence of APPV-specific antibodies (Abs) on established CSF serology, a novel indirect APPV-specific enzyme-linked immunosorbent assay (ELISA) was developed. For this purpose, the glycoprotein E^rns^ of the German APPV sequence S5/9 (GenBank NC_030653) was expressed in *Leishmania tarentolae* using the LEXSYcon2 Expression Kit (Jena Bioscience, Jena, Germany). The E^rns^ antigen was cloned into the pLEXSY-hyg2 plasmid in frame with the N-terminal LmSAP signal peptide for secretory expression and the C-terminal 6x His Tag for antigen purification. To confirm that the novel ELISA was specific for APPV, multiple samples of the reactive sera were analyzed via immunoblot using the purified E^rns^ protein. One specific protein band was detected in all tested sera and by the His-tag specific antibody (data not shown). Pig sera from Germany and Italy (*n*=189), that is, countries that are free of CSF and are not applying vaccination, were used to determine the APPV Ab statuses and the reactivities in two ELISAs targeting the E2 glycoprotein of CSFV (IDEXX, IDvet) and one pestivirus-specific ELISA targeting NS3 (BVDV/MD/BDV Ab ELISA, IDEXX). The APPV ELISA demonstrated an intermediate (S/P value=0.5–1) to high (S/P value>1.0) reactivity in 62% (118/189) of the investigated sera, which indicated high sero-prevalences in both countries. Independently of the APPV Ab status, all the tested sera were Ab-negative in the pestivirus ELISA and in one of the CSFV-specific ELISAs (IDEXX). Three doubtful and one false positive results in the other CSFV-specific ELISA (IDvet) were not correlated with the APPV Ab status. Accordingly, there is no evidence for serological cross-reactivity of the APPV Ab-positive samples with the ELISAs that are routinely applied for the detection of pestivirus- or CSFV-specific antibodies.

Attempts to isolate APPV from the cerebrospinal fluid of a CT-affected piglet were successful when using a porcine kidney cell line, as recently described.^8^ For virus isolation, 200 μL serum or cerebrospinal fluid from seven congenital tremor-affected piglets were used to inoculate SPEV cells (cell line 0008, Collection of Cell Lines in Veterinary Medicine, FLI, Germany) in a 24-well dish (37 °C, 72 h). The success of virus isolation was monitored via the APPV-specific SYBR Green based qRT-PCR of the cellular RNA as previously described.^3^ One of the fourteen inoculated wells was found to contain significant amounts of APPV genome (Cq value of 28) after the third passage, and this amount increased after repeated passages (Cq value of 19.8 in the 10th passage), which provided strong evidence for APPV replication. Immunostaining with a field serum that was found to be reactive against the recombinantly expressed E^rns^ antigen confirmed the presence of APPV-infected cells (Figure 1). Staining revealed foci of APPV-positive cells but did not reveal a continuous infection of the monolayer. The isolation of APPV allowed us to determine the specificity of the monoclonal antibodies (mAbs) that are routinely used for the detection of CSFV via immunostaining. The widely used mAbs 8.12.7 and C16 targeting the highly conserved NS3 are well known for their broad reactivity against various established pestivirus species, including CSFV, bovine viral diarrhea virus and border disease virus, and additional tentative pestivirus species from domestic and wild ruminants.^9, 10^ The mAb C16 is also listed in the technical part of the EU diagnostic manual. Despite the positive immunofluorescent staining of the APPV-infected cells with the APPV antibody-positive field serum, both of the pestivirus-specific mAbs were unable to recognize the APPV NS3 in the immunofluorescence application (Figure 1), although previous studies have demonstrated that NS3 is one of the most conserved regions among the polyproteins of APPV and CSFV and displays up to 60% similarity at the amino-acid sequence level.^3^ In addition, an APPV anti-serum was not suitable for staining SK6 cells that were infected with CSFV reference strain Alfort-Tuebingen (genotype 2.3).

CSFV-induced CT cannot be clinically distinguished from APPV-associated neurological disorders in newborn piglets. Nevertheless, the obtained data provide no evidence that the presence of APPV genomes or APPV-specific Abs interfere with the diagnostic tests that are routinely applied in the diagnosis of CSF. In conclusion, it seems unlikely that the newly discovered porcine pestivirus APPV will negatively influence the established CSF diagnosis and surveillance programs that are performed in many countries worldwide.

## Figures and Tables

**Figure 1:**
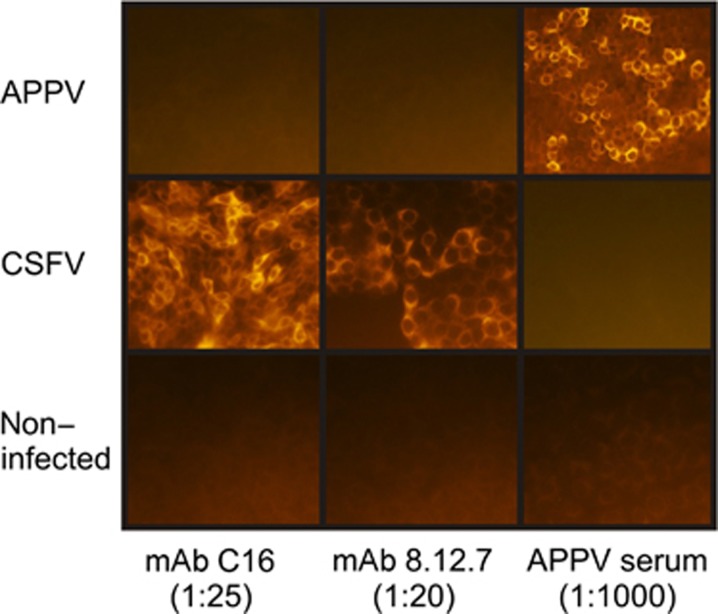
Immunostaining of APPV- and CSFV-infected porcine kidney cells. APPV genome-positive cells (11th passage of SPEV cells) were positively stained with a porcine field serum (diluted 1:1000; goat α-swine IgG, Cy3-labeled, Dianova, #114-165-003, 1:250) that was also reactive against the recombinantly expressed APPV E^rns^. The same passage of APPV-positive cells was not detected by the broadly reactive pestivirus NS3-specific mAb 8.12.7 (diluted 1:20; goat α-mouse IgG, Cy3-labeled, Dianova, #115-165-003, 1:800) or mAb C16 (diluted 1:25; goat α-mouse IgG, Cy3-labeled, Dianova, #115-165-003, 1:800). The APPV-specific field serum did not stain the CSFV-infected SK6 cells.
