# Supplementary material for: Recent emergence of a novel porcine pestivirus: interference with classical swine fever diagnosis?
Source: Emerg Microbes Infect. 2017 Apr 12;6(4):e19–. doi: 10.1038/emi.2017.5 (PMC5457672; doi:10.1038/emi.2017.5)
Supplement: Supplementary Table S1 [file emi20175x1.pdf]

Supplementary Table S1 Detection of APPV genomes by different qRT-PCRs.

		results of qRT-PCRs [Cq values]				
**Country**	**Region**	**APPV** (Postel, 2016)	**PanPesti** (this study)	**Pestivirus** (Becher, 1998)	**CSFV** (virotype RT-PCR kit, Qiagen)	**CSFV** (Hoffmann, 2005)
Germany	NRW	22.1	25.5	-	-	-
Germany	NRW	16.3	20.6	-	-	-
Germany	NRW	20.0	24.2	-	-	-
Germany	NRW	22.3	25.4	-	-	-
Germany	BW	23.9	27.1	-	-	-
Germany	BW	35.0	-	-	-	-
Germany	Bavaria	30.5	28.0	-	-	-
Germany	Bavaria	34.8	-	-	-	-
Germany	Lower Saxony	28.6	23.8	-	-	-
Germany	Lower Saxony	32.6	26.1	-	-	-
Italy	Umbria	24.9	26.5	-	-	-
Italy	Umbria	30.6	-	-		-
Italy	Umbria	24.9	31.6	-	-	-
Italy	Umbria	21.3	23.7	-	-	-

Cq, Quantitation cycle; NRW, North Rhine-Westphalia; BW, Baden-Württemberg
